# Codon optimization and factorial screening for enhanced soluble expression of human ciliary neurotrophic factor in *Escherichia coli*

**DOI:** 10.1186/s12896-014-0092-x

**Published:** 2014-11-14

**Authors:** Jaakko M Itkonen, Arto Urtti, Louise E Bird, Sanjay Sarkhel

**Affiliations:** Centre for Drug Research, Division of Pharmaceutical Biosciences, Faculty of Pharmacy, University of Helsinki, P.O. Box 56, Viikinkaari 5E, Helsinki, 00014 Finland; School of Pharmacy, University of Eastern Finland, Kuopio, Finland; Division of Structural Biology, Henry Wellcome Building for Genomic Medicine, University of Oxford, Roosevelt Drive, Oxford, OX3 7BN UK; OPPF-UK, Research Complex at Harwell, R92 Rutherford Appleton Laboratory, Harwell Oxford, Didcot Oxford, OX11 OFA UK

**Keywords:** Neurotrophic factors, Human CNTF, Codon optimization, Recombinant soluble expression, *E. coli*

## Abstract

**Background:**

Neurotrophic factors influence survival, differentiation, proliferation and death of neuronal cells within the central nervous system. Human ciliary neurotrophic factor (hCNTF) has neuroprotective properties and is also known to influence energy balance. Consequently, hCNTF has potential therapeutic applications in neurodegenerative, obesity and diabetes related disorders. Clinical and biological applications of hCNTF necessitate a recombinant expression system to produce large amounts of functional protein in soluble form. Earlier attempts to express hCNTF in Escherichia coli (*E. coli*) were limited by low amounts and the need to refold from inclusion bodies.

**Results:**

In this report, we describe a strategy to effectively identify constructs and conditions for soluble expression of hCNTF in *E. coli*. Small-scale expression screening with soluble fusion tags identified many conditions that yielded soluble expression. Codon optimized 6-His-hCNTF construct showed soluble expression in all the conditions tested. Large-scale culture of the 6-His-hCNTF construct yielded high (10 – 20 fold) soluble expression (8 – 9 fold) as compared to earlier published reports. Functional activity of recombinant 6-His-hCNTF produced was confirmed by its binding to hCNTF receptor (hCNTFRα) with an EC_50_ = 36 nM.

**Conclusion:**

Our results highlight the combination of codon optimization and screening soluble fusion tags as a successful strategy for high yielding soluble expression of hCNTF in *E. coli*. Codon optimization of the hCNTF sequence seems to be sufficient for soluble expression of hCNTF. The combined approach of codon optimization and soluble fusion tag screen can be an effective strategy for soluble expression of pharmaceutical proteins in *E. coli*.

**Electronic supplementary material:**

The online version of this article (doi:10.1186/s12896-014-0092-x) contains supplementary material, which is available to authorized users.

## Background

Neurotrophic factors play central role within the nervous system. They regulate cell survival, proliferation, differentiation, migration, dendrite and axonal growth, synaptic plasticity and interactions of neuronal and glial cells [1–4]. The neuropoietic cytokine, ciliary neurotrophic factor (CNTF), first identified in chick ciliary neurons [5,6], belongs to the interleukin-6 and leukemia inhibitory factor (LIF) family of four helix bundle cytokines [7,8]. Human CNTF (hCNTF), a polypeptide of approximate 23 kDa, is involved in the maintenance of ciliary and motor neurons. hCNTF exerts its signaling effect by binding to a heterotrimeric complex of hCNTF receptor (hCNTFRα), gp130 and LIF receptor (LIFRβ) [9–11]. Apart from neuroprotection [12,13], hCNTF is also known to influence energy balance [14–16]. Consequently, hCNTF has potential therapeutic applications not only in neurodegenerative diseases such as Amytropic Lateral Sclerosis (ALS) and Huntington’s disease (HD), but also in obesity and related type II diabetes. hCNTF secreting implant has completed phase II studies as an effective therapy for dry age related macular degeneration (AMD) in the eye [17].

The therapeutic potential of CNTF necessitates a recombinant production system for large amounts of protein for clinical and biological applications. Bacterial expression systems are known to be rapid and economical for obtaining recombinant proteins [18–22]. However, attempts to express hCNTF in *Escherichia coli* (*E. coli*) have either yielded low soluble levels (approx. 13%) or insoluble inclusion bodies [8,23–26]^a^. Soluble recombinant expression of pharmaceutical proteins is of utmost importance not only for direct applications but to be able to employ advanced molecular engineering methods to alter their physicochemical properties. Bacterial expression systems offer several advantages; they are relatively inexpensive to culture, can be easily modified genetically and have fast turnaround time. *E. coli*, a preferred bacterial host, is considered to be a ‘laboratory workhorse’ for recombinant protein expression. However, the advantages are easily offset by the challenges poised by recombinant production of eukaryotic proteins [27,28]. Bacterial hosts such as *E. coli* are often limited in amounts of tRNA for the codons that are used rather less frequently. Instances where codon bias of the gene is significantly different from that of the expression host *E. coli*, paucity of the tRNAs severely limits the translation process. This results in non-optimal translation of the RNA including stalling, termination, possible frame-shifting and low levels of protein expression [29,30]. Apart from codon usage being a determining factor in protein expression levels, structural features of mRNA that interfere with the initiation of translation is also believed to adversely affect protein expression [31,32]. In contrast, a recent study found that there was very less influence of mRNA structure on protein expression and that depletion of charged tRNAs was the main limiting factor for protein expression [33]. Codon optimization of the target gene and/or use of tRNA enhanced strains have become an attractive starting point for heterologous protein expression in *E. coli*. Codon optimization has been successfully utilized to express human pigment epithelium derived factor in *E. coli* [34] and thirty human short chain dehydrogenase/reductive genes showed vast improvement in expression with optimized codon and use of *E. coli* strains containing rare codon tRNAs [35]. The other relevant issue with heterologous expression in *E. coli* is the formation of unordered aggregates (inclusion bodies) due to improper folding of the polypeptide chain. High rates of protein expression or unfavorable conditions for protein folding cause inclusion bodies formation. Successful strategies to mitigate improper folding include decreasing the cultivation temperature [36], co-expression of folding modulators [37] and reducing the rate of gene expression [38]. An alternative strategy to improve the solubility has been to express the protein of interest fused to a solubility enhancing polypeptide ‘tag’ [39–42]. The soluble ‘tag’ also serves to provide a reliable translation initiation [43]. Although useful, the fusion ‘tag’ strategy is limited by the lack of a rational design for choice and a given ‘tag’ might be effective with only certain targets [40,44–46]. In general, production of soluble proteins in *E. coli* remains largely a trial-and-error process.

In this study, we report an effective strategy towards screening and optimization for soluble expression of hCNTF in *E. coli*. We address the twin issues of codon bias and possible improper folding by optimizing the hCNTF gene sequence for use in *E. coli* and adopting a factorial screening strategy. Small-scale screening methods have been successful in structural genomics initiatives [47–49] in providing reproducible qualitative and quantitative leads for large-scale expression. hCNTF gene was codon optimized and cloned into a suite of nine vectors harboring different fusion (soluble/affinity) tags. The constructs were screened for expression in two different *E. coli* strains and culture medias at different temperatures.

## Results and discussion

The objective of our work was to achieve soluble, high yielding production of hCNTF in *E. coli*. Earlier attempts of hCNTF production in *E. coli* resulted in low amounts of soluble protein or required purification from insoluble inclusion bodies [8,23-26]. McDonald and co-workers have reported the presence of only 13% of soluble hCNTF in *E. coli* (BL21) cell extracts [23]. In another report, the insoluble fraction from bacterial cell lysate was found to contain 80% of recombinant hCNTF [24]. Masiakowski et al. have also reported purifying hCNTF from inclusion bodies even though the translation of recombinant hCNTF was 20 – 40% of total protein [25]. Purification of hCNTF from inclusion bodies requires an additional refolding step and such processes are usually not desirable since they are cumbersome and in many cases might result in non-native state of the target protein. Non-native state of proteins often leads to aggregation that can cause fatal immunogenic reactions from protein therapeutics [50]. Thus, for proteins of pharmaceutical relevance, such as hCNTF, it is highly desirable to have high yielding, soluble and cost effective production of protein. An effective strategy to express soluble proteins requires cost effective screening system where several factors could be tested in parallel. Structural genomics initiatives have effectively utilized such approaches to produce recombinant proteins in *E. coli* [47–49].

**Figure 1:**
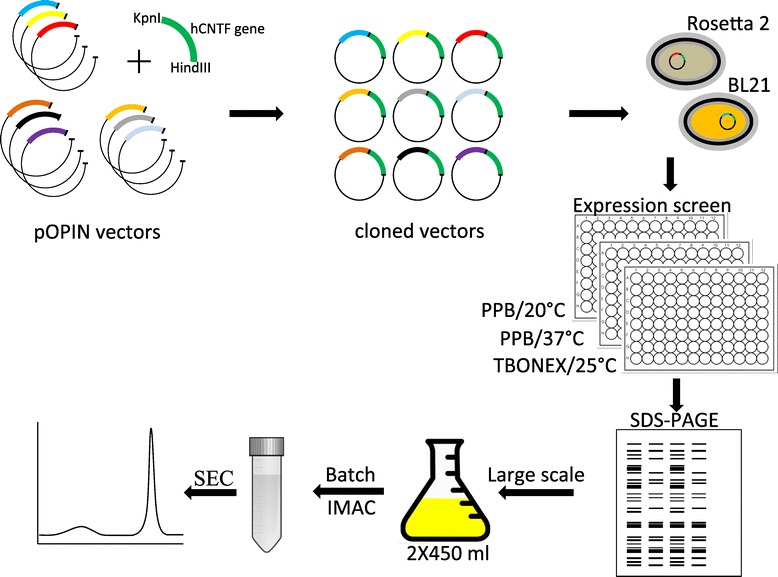
**Scheme depicting the overall strategy of parallel cloning, small-scale expression screening and large-scale hCNTF production.**

**Figure 2:**
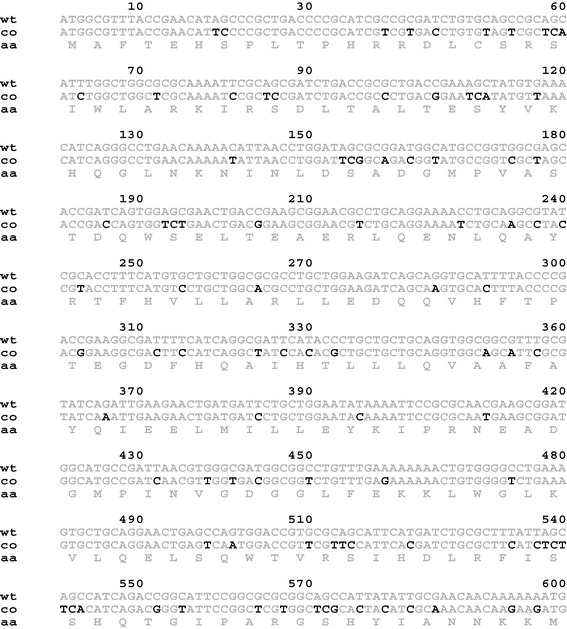
**Comparison of wild type (wt) and codon optimized (co) hCNTF sequence.**

In our present work, we adopted the strategy of fusing the codon optimized sequence of hCNTF to nine tags (eight soluble tags and the 6–His tag) and carrying out a factorial screening for protein expression in different conditions (temperature/culture media/expression strains). The overall scheme is depicted in Figure 1. The codon optimized hCNTF sequence is shown in Figure 2. A proprietary algorithm (OptimumGene™, Genscript USA) from the vendor was used to increase the codon usage bias in *E. coli* by improving the codon adaptation index (CAI) to 0.87. GC content and unfavorable peaks were optimized to prolong the half-life of the mRNA. Stem-loop structures that have an impact on ribosomal binding and stability of mRNA, were disrupted. In addition, the codon optimization process screened and successfully modified negative *cis*-acting sites. The construction of the pOPIN family of vectors have been described elsewhere [51–53]. Each vector has a N-terminal fusion ‘tag’ (Table 1) followed by Rhinovirus 3C protease cleavage site between the ‘tag’ and the inserted target sequence to facilitate ‘tag’ removal. The set of vectors have common cloning sites (KpnI and HindIII) that facilitate parallel cloning and construction of expression vectors. A cloning efficiency of 100% was achieved by picking three clones for each vector construction. The constructs were transformed into *E. coli* strains, BL21(DE3)pLysS and Rosetta 2(DE3)pLysS (a derivative of BL21 supplying tRNAs for 7 rare codons; AGA, AGG, AUA, CUA, GGA, CCC, and CGG) and small-scale expressions were carried out with culture medias Power Prime Broth (PPB) at 20°C/37°C and Overnight Expression Terrifc Broth (TBONEX) at 25°C.

**Table 1:** ***Expression vectors and their fusion tags***

**Lane**	**Vector**	**Fusion tag**	**BL21**			**Rosetta 2**		
			**20°C PPB**	**37°C PPB**	**25°C TBONEX**	**20°C PPB**	**37°C PPB**	**25°C TBONEX**
1	pOPIN**F**	6-His-	1	1	1	1	1	1
2	pOPIN**S3C**	6-His-SUMO-	0.7	1.5	2.1	1.3	2.7	1.1
3	pOPIN**TRX**	6-His-Thioredoxin-	1.2	1.7	4.9	1.1	0.7	0.1
4	pOPIN**MSYB**	6-His-MsyB-	0.5	0.8	2.9	1.8	3.6	2.0
5	pOPIN**J**	6-His-GST-	0.3	1.3	0.4	0.1	0.5	0.1
6	pOPIN**HALO7**	6-His-Halo-	1.5	1.6	1.7	1.6	3.0	2.0
7	pOPIN**M**	6-His-MBP-	0.8	1.4	2.5	0.9	1.1	0.7
8	pOPIN**TF**	6-His-Trigger Factor-	0.5	1.1	1.7	0.3	0.6	0.5
9	pOPIN**NusA**	6-His-NusA-	0.6	1.2	1.1	0.7	0.8	0.1

*Empirical scores of soluble fraction yield in small-scale expression relative to 6-His-construct. Highlighted text in bold within the vector name symbolize the fusion tag.*

The soluble fusion tags used in expression screening are listed in Table 1. The choice of soluble tags has been dictated by literature precedence on the success rate of soluble expression of a diverse set of proteins. No single soluble tag can increase the expression and solubility of all target proteins. However, some general trends on effectiveness of a limited set of tags (amongst those used in present study) have been reported in some studies. SUMO and NusA were found to be equally efficient in terms of total and soluble protein expression in studies on three model proteins [41,45]. The success of a particular soluble fusion tag is dependent on the target protein of interest and hence the need to screen a diverse set. Both the culture medias used, PPB and TBONEX, are rich and support high cell densities. TBONEX, being an auto-induction media, also offers the advantage of a simplified expression protocol by eliminating the monitoring and induction steps. Induction free protocol is particularly useful for high-throughput protein expression approaches. Culture growth in PPB was screened at temperatures 20°C and the normal 37°C. Lower culture temperature is known to facilitate proper folding and soluble protein expression [36].

**Figure 3:**
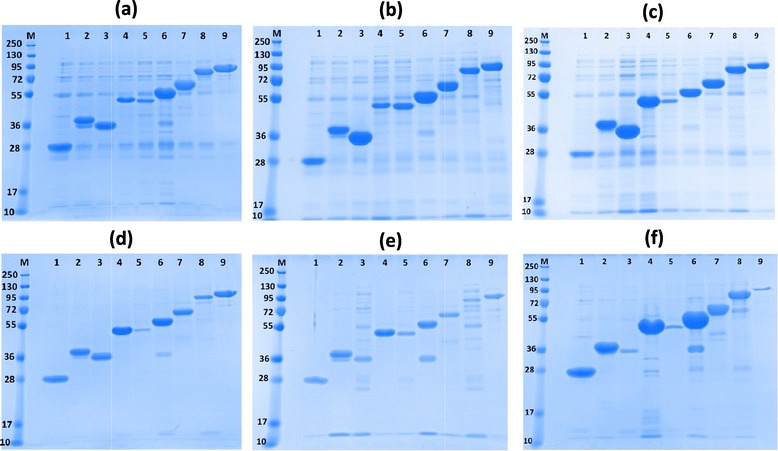
**SDS-PAGE analysis of small-scale expression screen. (a)** BL21/PPB/20°C; **(b)** BL21/PPB/37°C; **(c)** BL21/TBONEX/25°C; **(d)** Rosetta 2/PPB/20°C; **(e)** Rosetta 2/PPB/37°C; **(f)** Rosetta/TBONEX/25°C. Lanes M, 1–9 denote protein marker and Ni-NTA purified soluble hCNTF fusion constructs from expression vectors as listed in Table 1, respectively. The gel was stained with Coomassie Blue dye.

The soluble fractions were treated with Ni-NTA magnetic beads to capture recombinant 6-His-(fusion tag)-hCNTF proteins. The eluted soluble, Ni-NTA affinity purified fractions were analyzed on a SDS-PAGE gel (Figure 3).

**Figure 4:**
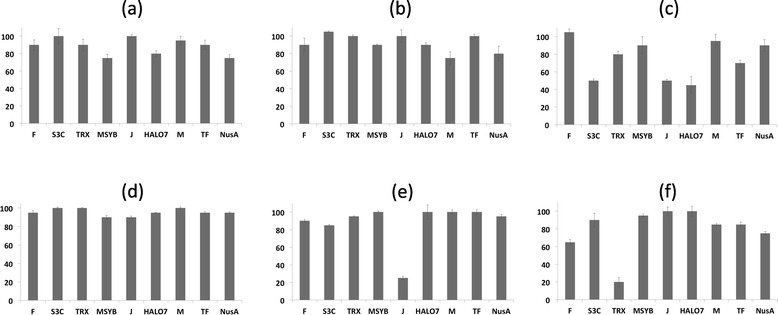
**Histograms depicting percentage soluble fraction of hCNTF in small-scale expression screen. (a)** BL21/PPB/20°C; **(b)** BL21/PPB/37°C; **(c)** BL21/TBONEX/25°C; **(d)** Rosetta 2/PPB/20°C; **(e)** Rosetta 2/PPB/37°C; **(f)** Rosetta/TBONEX/25°C. The estimates are based on measurements from three technical repeats.

Most conditions (constructs/temperature/media) screened for expression showed the presence of soluble hCNTF as fusions (Figures 3 and 4). On an average, the percentage of hCNTF in the soluble fraction was close to 80% or more in majority of the conditions screened. Relative amounts of soluble hCNTF as fusions with soluble tags were empirically estimated (comparing intensity of bands) with respect to the 6-His-construct in each experimental condition (Table 1). The intensity of the bands depends on protein concentration and also on the size and content of residue types of the construct. The empirical scores are not normalized against molecular size and residue content and as such the scores should not be used for direct comparisons but for easy visual summarization of relative trends. The efficacies of the fusion tags (scores averaged over the six expression conditions) were ranked as compared to the 6-His- tag. The trend observed was in the order of MsyB/Halo (1.9-fold); SUMO/Trx (1.6-fold); MBP/TF/NusA (approx. 1-fold) and GST (0.5 fold). Significant proteolysis seems to have taken place for some of the larger fusions (Figure 3; Halo, MBP, TF). This could possibly be due to improper folding of the larger fusion constructs. In a recent publication [53], Bird has reported similar observations with the larger fusion constructs. The most significant observation of our small scale screening experiment was that under all conditions, 6-His-hCNTF constructs showed appreciable amounts of expression. It is highly desirable to obtain soluble expression of proteins without a solubility enhancing fusion partner as the downstream purification process can avoid additional steps of tag cleavage and protease removal. Also, some proteins might exhibit markedly low solubility after the fusion tag has been cleaved off; limiting the utility of soluble fusion tags in such cases. The 6-His-tag is merely an affinity tag for purification and in all probability does not improve the solubility of recombinant hCNTF as such, unlike the other fusion tags. In fact, in a study on 20 human proteins, the 6-His-tag had a negative impact on protein solubility when present at either the N- or C-terminus [54]. In this context, our results for soluble expression of 6-His-hCNTF assume significant importance. Assuming that the 6-His-tag does not influence the solubility of recombinant hCNTF, or at least in a positive way, the soluble expression of hCNTF in the conditions tested can be attributed to codon optimization of the hCNTF sequence. Our experimental design was aimed at maximizing the chances of obtaining soluble protein in the backdrop of literature precedence [23–26]. However, we do note that in earlier published reports [23–26] of recombinant expression in *E. coli* with the wild type hCNTF; conditions of expression as varied as in our experimental set up (soluble fusion tags, lower temperatures and expression strain rich in tRNAs for rare codons) were not explored. Results from the earlier studies hint at the fact that most probably both translation (low yields of 5–10 mg/L) and folding (inclusion body formation) were the limiting factors in expression. It might thus be of interest to see the outcome of recombinant expression of the wild type sequence in a strain rich in tRNAs for rare codons, such as Rosetta 2(DE3) or as a fusion construct with soluble tags in a strain, such as BL21(DE3). Even if the results of such experiments were positive, the codon optimized sequence used in the present study clearly results in a vast improvement since it allows high yielding soluble expression in BL21 at 37°C (approx. 90% or more in soluble fraction) without the aid of soluble fusion tags in contrast to the wild type sequence under similar conditions in previous studies [23–26]. With easy and cheap availability of synthetic genes, the optimized hCNTF codon sequence presents an ideal option for recombinant production in BL21 (a common laboratory strain) without the need for a soluble fusion tag.

**Figure 5:**
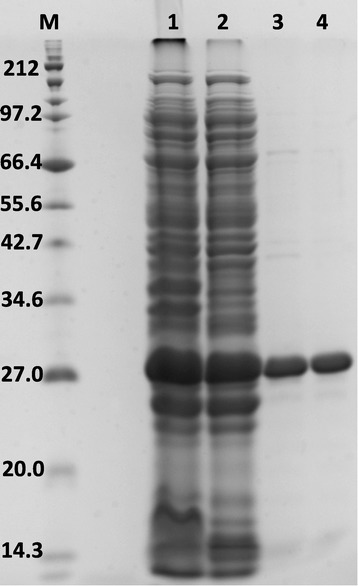
**SDS-PAGE analysis of recombinant 6-His-hCNTF purification.** M: protein marker; 1: crude cell lysate; 2: soluble fraction; 3: Ni-IDA purified hCNTF; 4: size exclusion chromatography purified hCNTF. The gel was stained with Coomassie Blue dye.

Results from small-scale expression show varying efficacies of the fusion constructs in different conditions of growth as determined empirically (Table 1). However, since the overall yield after the cleavage of the fusion tag and purification might be very different from that of the fusion construct and also the need for additional tag cleavage and removal steps; 6-His-hCNTF was chosen for large scale expression with Rosetta 2(pLysS) and the auto inducing media, TBONEX at 25°C as a representative example. The analysis of the overall purification scheme is shown in Figure 5.

**Figure 6:**
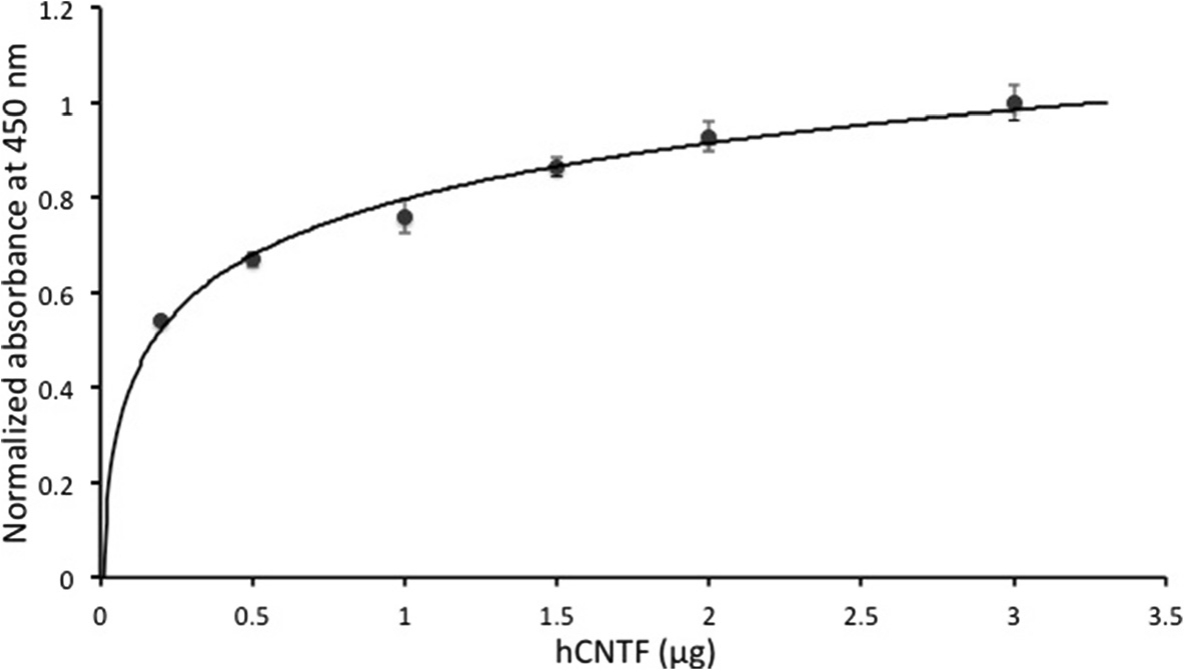
**Binding of biotinylated hCNTF to hCNTFRα.**

The amount of hCNTF protein obtained after final purification was 112 mg/L of culture. This is a vast improvement (greater than 10 – 20 fold) in overall yield from earlier reports of 5 – 10 mg/L [23,24]. Functional activity of recombinant hCNTF was confirmed by binding to hCNTFRα (Figure 6). The binding showed an EC_50_ value of 36 nM in accordance with an earlier published report [55]. A very recent report [56] published during the course of this manuscript preparation, has highlighted an attractive alternate strategy to express high yielding soluble hCNTF in *E. coli*. The study reports the usage of a single protein production (SPP) system that co-expresses *E. coli* toxin mRNA interferase, MazF and hCNTF. The strategy targets down-regulating the expression of endogenous bacterial proteins by selectively cleaving the ACA sites in host mRNAs by MazF. The hCNTF gene sequence was optimized for codon usage in *E. coli* and removing the ACA sites. So, whereas in the recently published study, a codon optimization strategy was directed at more specific use with the expression system, in our present study, a more sophisticated proprietary codon optimization algorithm OptimumGene™ (GenScript, NJ, USA) was used. hCNTF codons in our present study were not only optimized for codon usage in *E. coli* but parameters such as GC content, CpG dinucleotides content, mRNA secondary structure, cryptic splicing sites, premature PolyA sites, internal chi sites and ribosomal binding sites, negative CpG islands, RNA instability motif, repeat sequences were also optimized. The SPP system seems to be a promising approach, however, its robustness as a general strategy needs to be validated for a number of proteins. It appears that the SPP system might be useful in obtaining high yields of recombinant proteins indirectly by suppressing endogenous protein expression. However, protein sequences that might not fold properly once translated might still need soluble tags for proper folding. From such a perspective, our approach to screen with a suite of soluble tags might have a more versatile utility. It would be interesting to integrate our approach (a sophisticated codon optimization and screening with soluble tags in different strains of *E. coli*) with the SPP system (down-regulating endogenous bacterial proteins) for enhanced expression of difficult eukaryotic targets.

## Conclusions

We report here high yielding soluble expression of functional hCNTF in *E. coli*. Our combined approach of codon optimization and screening for soluble expression with nine fusion tags under varying culture conditions identified several constructs producing soluble hCNTF expression (8 – 9 fold higher soluble fraction) and the overall yield of purified recombinant hCNTF achieved was significantly higher (greater than 10 – 20 fold) as compared to previous published reports. Soluble expression of the 6-His-hCNTF in all conditions tested points to the fact that codon optimization is sufficient for soluble expression of hCNTF in *E. coli*. However, for maximal yields, some fusion constructs might be more useful than 6-His-hCNTF. The strategy of combining codon optimization and fusion tag screening might be useful for soluble expression of pharmaceutical proteins in *E. coli*.

## Methods

### Codon optimization

The codons of the hCNTF gene were optimized for optimal usage in *E. coli* using a commercial proprietary algorithm, OptimumGene™ (GenScript, NJ, USA). Variety of parameters were optimized, including codon usage bias, GC content, CpG dinucleotides content, mRNA secondary structure, cryptic splicing sites, premature PolyA sites, internal chi sites and ribosomal binding sites, negative CpG islands, RNA instability motif, repeat sequences. The synthetic gene was purchased from GenScript, US.

### Construction of expression vectors

The construction of pOPIN-fusion vector backbones has been described elsewhere [51–53]. The complete coding sequence of hCNTF was synthesized by GenScript, NJ, USA and provided as an insert within pUC57 plasmid. hCNTF gene was amplified by polymerase chain reaction (PCR) using KOD Hot Start (Novagen) with primers that had 5’-extensions of AAGTTCTGTTTCAGGGCCCG and ATGGTCTAGAAAGCTTTA on the forward and reverse primers, respectively (Additional file 1: Figure S1). The set of nine vectors harboring various fusion tags is listed in Table 1. The purified PCR product was inserted into digested pOPIN vectors between KpnI and HindIII restriction sites using Gibson Assembly™ cloning kit (New England Biolabs, MA, USA) and the reaction mixture transformed into NEB 5α (New England Biolabs, MA, USA) competent cells. Three colonies were screened for each construct and the presence of insert verified by PCR using a vector specific forward primer and hCNTF gene specific reverse primer (Additional file 1: Figure S2). pOPIN expression vectors 6-His-(fusion tag)-hCNTFs obtained were (6-His)-hCNTF, 6-His-(S3C)-hCNTF, 6-His-(Trx)-hCNTF, 6-His-(MSYB)-hCNTF, 6-His-(J)-hCNTF, 6-His-(HALO)-hCNTF, 6-His-(M)-hCNTF, 6-His-(TF)-hCNTF, 6-His-(NusA)-hCNTF.

### Small-scale expression, affinity purification for soluble protein characterization

Expression strains BL21 (DE3)pLysS (Novagen) and Rosetta 2(DE3)pLysS (Novagen) were transformed with expression plasmids. Single colonies were used to inoculate 0.7 ml Power Prime Broth, PPB (AthenaEs) supplemented with ampicillin (100 μg/ml) and chloramphenicol (34 μg/ml) taken in a 2 ml 96-well plate. The plates were sealed with gas permeable adhesive seals and the seed cultures grown overnight at 37°C with shaking at 250 rpm. For the small-scale expression screen, 150 μl of BL21(DE3)pLysS and 250 μl of Rosetta 2(DE3)pLysS seed cultures were used to inoculate 3 ml of PPB and Overnight Express™ Instant TB media, TBONEX (Novagen) taken in a 10 ml 24-deepwell plate and supplemented with ampicillin (100 μg/ml) and chloramphenicol (34 μg/ml). Cultures in PPB were grown at 37°C with shaking at 250 rpm. For expression at 37°C, 1 mM IPTG was added when the OD_600_ reached between 0.5 - 0.6. For expression at 20°C, the cultures were cooled for 15 minutes on a bench top before adding 1 mM IPTG and grown at 20°C overnight (18 h) with shaking at 250 rpm. TBONEX auto-inducing cultures were grown at 25°C for 24 h with shaking at 225 rpm. The cultures were harvested by centrifugation and the supernatant removed from the pellets. The pellets were stored at −80°C. The frozen pellets were resuspended in 210 μL of 50 mM NaH_2_PO_4_, 300 mM NaCl, pH 8.0; 10 mM imidazole, 1 mM dithiothreitol (DTT), protease inhibitor cocktail, PIC (Sigma). The pellets were freeze-thawed three times; resuspending each time in between the freeze thaw cycle. 1 μL of Lysonase (EMD Millipore) was added to each pellet suspension and incubated at room temperature with shaking at 270 rpm for 45 minutes. The lysates were then centrifuged at 21,100 g at 4°C for 40 minutes. 20 μL of suspended nickel nitriloacetic (Ni-NTA) magnetic beads (GenScript, NJ, USA) was dispensed into each well of a 96-well flat bottom plate. The beads were conditioned with 50 mM NaH_2_PO_4_, 300 mM NaCl, pH 8.0; 10 mM imidazole, 1% Tween 20. Supernatants from cleared lysates were transferred to the wells containing the magnetic beads and incubated at room temperature for 50 minutes with shaking at 270 rpm. The plate was then placed on a magnetic rack (New England Biolabs, MA, USA) for 5 minutes and the supernatant was removed. The magnetic beads were washed four times with 50 mM NaH_2_PO_4_, 300 mM NaCl, pH 8.0; 20 mM imidazole, 0.05% Tween 20 and 1 mM DTT. Proteins bound to the magnetic beads were eluted by adding 50 μL of 50 mM NaH_2_PO_4_, 300 mM NaCl, pH 8.0; 250 mM imidazole, 0.05% Tween 20 and 1 mM DTT and incubating with shaking (270 rpm) at room temperature for 10 minutes. The plate was placed on the magnetic rack for 7 minutes and 45 μL of the supernatant was removed. Protein samples were heated at 95°C with SDS-PAGE sample buffer and analyzed on a SDS-PAGE gel. The 6-His-fusion constructs of hCNTF were verified by specific detection of the 6-His tag using Pierce 6XHis protein tag stain reagent set (Thermo Fisher Scientific Inc., USA).

### Estimation of soluble fraction of total recombinant protein expressed

Cell pellets from 3 ml expression culture were thawed and resuspended in 250 μL of 50 mM NaH_2_PO_4_, 300 mM NaCl pH 8.0; 0.2% Tween 20. The pellets were freeze-thawed three cycles and 50 μL of the suspended pellets were each treated with 1 μL of Lysonase. The pellets were incubated at room temperature for 45 minutes with shaking at 270 rpm. The mixture was divided into two fractions of 25 μL each. For total protein samples, 25 μL of the cell suspension was diluted with an equal volume of water followed by 10 μL of 6X SDS-PAGE sample buffer and boiled for 15 minutes at 95°C. The samples were centrifuged at high speed for 15 minutes and the supernatant run as the ‘total protein’ sample on a SDS-PAGE gel. For the soluble fraction, the 25 μL cell suspension was diluted with an equal volume of water and centrifuged at high speed for 15 minutes. The supernatant was removed and mixed with 10 μL of 6X denaturing SDS-PAGE sample buffer and boiled for 15 minutes at 95°C. The samples were analyzed as ‘soluble fraction’ on a SDS-PAGE gel. GelQuant.NET software (V 1.7.8) from biochemlabsolutions.com was used to quantitate band intensities to estimate the ratio of soluble to total protein. The values were averaged over three measurements. The intensity estimates for comparisons between constructs should be restricted to samples run within the same gel.

### Large-scale expression and purification of hCNTF

6-His-hCNTF construct was chosen for large scale expression in Rosetta 2 (DE3)pLysS with the auto-inducing media TBONEX. 2 X 450 ml of cultures were inoculated with overnight seed cultures (30:1) and grown at 25°C with shaking at 270 rpm for 30 h. The cells were harvested by centrifugation at 10,000 g for 30 minutes at 4°C. and frozen at −80°C. The cell pellet was resuspended in lysis buffer (50 mM NaH_2_PO_4_, 300 mM NaCl, pH 8.0; 10 mM imidazole, 1 mM DTT and PIC) and freeze thawed three cycles, resuspending thoroughly after thawing each time. Lysonase was added to the suspension and incubated at room temperature for 60 minutes with gentle agitation. The lysate was centrifuged at 14,500 g at 4°C for 50 minutes and the cleared supernatant incubated with Ni-IDA resin (Macherey Nagel) at 4°C for 120 minutes with gentle agitation. The supernatant was removed by centrifugation at 500 g for 3 minutes and the Ni-IDA resin washed with 50 mM NaH_2_PO_4_, 300 mM NaCl, pH 8.0; 10 mM imidazole, 1 mM DTT and PIC. In subsequent washes, the imidazole concentration was increased. Bound hCNTF was eluted out of the resin (Additional file 1: Figure S3) by adding 250 mM imidazole to the buffer and the eluted fractions pooled, concentrated and buffer exchanged in 100 mM NaH_2_PO_4_, 300 mM NaCl, pH 8.0; 1 mM DTT using Amicon® Ultra-4 centrifugal filter units with a 3 kDa molecular weight cut off (Merck Millipore). The Ni-IDA eluted protein was further purified by size exclusion chromatography (SEC) by loading onto a HiLoad Superdex 200 prep grade filtration column (Additional file 1: Figure S4). The protein fractions were pooled, concentrated and buffer exchanged in 100 mM NaH_2_PO_4_, 50 mM NaCl, pH 8.0; 2 mM DTT using centrifugal filter units.

### Binding assay of biotinylated hCNTF with hCNTFRα

hCNTF was labeled with biotin for binding studies with hCNTFRα. Briefly, hCNTF was reacted with a 20-fold molar excess of NHS-PEG4-biotin in a 100 mM carbonate buffer solution for 2 h at room temperature with gentle agitation. The reaction mixture was buffer exchanged with 100 mM NaH_2_PO_4_, 50 mM NaCl, pH 8.0 and concentrated using a spin filter having 3 kDa molecular weight cut off.

For the binding study, 1 μg of hCNTFRα (Sino Biologicals Inc.) in PBS was immobilized on the surface of a Nunc MaxiSorp flat-bottomed 96-well plate. The wells were washed with 50 mM Tris–HCl, 150 mM NaCl, 0.05% Tween 20, pH 7.0 (TBS-T) and blocked with a 2% BSA in TBS-T solution. After washings, biotinylated CNTF was added in varying amounts (0.2 – 3.0 μg) to the wells and incubated at room temperature for 2 h with gentle agitation. The wells were then washed with TBS-T followed by the addition of 50 ng of HRP (horseradish peroxidase)-conjugated streptavidin (Thermo Scientific) to each well and incubated at room temperature for 70 minutes with gentle agitation. The wells were washed and 200 μL of TMB (3,3’, 5,5’-tetramethylbenzidine; Thermo Scientific) was added to each well. The reaction was stopped with 2 M H_2_SO_4_ and the optical density measured at 450 nm using a spectrophotometer (Varioskan Flash; Thermo Scientific).

### Availability of supporting data

The data set supporting the results of this article are included within the article and in Additional file 1.

Additional file 1: Figures S1 - S4. Description: PCR amplified hCNTF, validation of hCNTF clones in expression vectors, affinity batch purification fractions, size exclusion chromatography profile.

### Endnote

^a^During the course of this manuscript preparation, an alternate expression system expressing soluble hCNTF in *E. coli* was published. This has been discussed in the ‘Results and Discussion’ section.

## Additional file

Additional file 1: Figure S1 PCR-amplified hCNTF insert. **Table S1** PCR primers for the amplification of the synthesized hCNTF. **Figure S2** PCR validation of cloned hCNTF in pOPIN vectors. **Figure S3** Representative eluted fractions from Ni-IDA batch purification of 2 × 450 ml cultures **(A and B)**. **Figure S4** Size exclusion chromatography (SEC).

## Abbreviations

hCNTF: Human ciliary neurotrophic factor
E. coli: Escherichia coli
hCNTFRα: Human ciliary neurotrophic factor receptor alpha
LIFβ: LIF receptor
ALS: Amytropic lateral sclerosis
HD: Huntington’s disease
AMD: Age related macular degeneration
tRNAs: Transfer RNAs
CAI: Codon adaptation index
TBONEX: Overnight expression terrific broth
PCR: Polymerase chain reaction
6-His: 6-histidine
SUMO: Small ubiquitin related modifier
Trx: Thioredoxin
MsyB: *E. coli* acidic protein
GST: Glutathione S-transferase
Halo: Mutated dehalogenase
MBP: Maltose binding protein
TF: Trigger factor
NusA: N-utilization substance A
DTT: Dithiothreitol
Ni-NTA: Nickel nitriloacetic
PPB: Power prime broth
IPTG: Isopropyl β-D-1-thiogalactopyranoside
SDS-PAGE: Sodium dodecyl sulphate polyacrylamide gel electrophoresis
PIC: Protease inhibitor cocktail
Ni-IDA: Nickel iminodiacetic acid
SEC: Size exclusion chromatography
PBS: Phosphate buffered saline
NHS-PEG4: N-hydroxysuccinimide ester – polyethylene glycol
TBS-T: Tris buffered saline-tween
HRP: Horseradish peroxidase
TMB: 3,3’, 5,5’-tetramethylbenzidine
SPP: Single protein production
