# Supplementary material for: Codon optimization and factorial screening for enhanced soluble expression of human ciliary neurotrophic factor in Escherichia coli
Source: BMC Biotechnol. 2014 Nov 14;14:92. doi: 10.1186/s12896-014-0092-x (PMC4237735; doi:10.1186/s12896-014-0092-x)
Supplement: Additional file 1: Figure S1 — PCR-amplified hCNTF insert. Table S1 PCR primers for the amplification of the synthesized hCNTF. Figure S2 PCR validation of cloned hCNTF in pOPIN vectors. Figure S3 Representative eluted fractions from Ni-IDA batch purification of 2 × 450 ml cultures (A and B). Figure S4 Size exclusion chromatography (SEC). [file 12896_2014_92_MOESM1_ESM.pdf]

SUPPLEMENTARY FIGURES

Figure S1

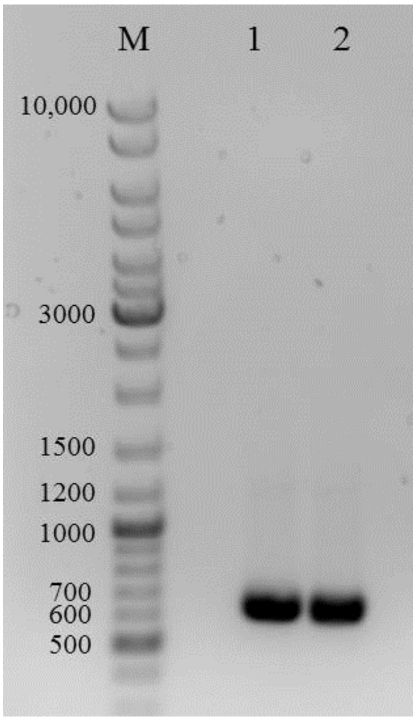

Fig. S1. PCR-amplified hCNTF insert. M denotes DNA marker (bp)**;** lanes 1 and 2 loaded with 2,5 µl of PCR-reaction mixture; lane 2: reaction carried out in presence of 3 % DMSO.

Table S1. PCR primers for the amplification of the synthesized hCNTF. fp, rp denote forward and reverse primers.

Primer	Sequence (5' → 3')
CNTF_1_fp	AAGTTCTGTTTCAGGGCCCGATGGCGTTTACCGAACATTCC
CNTF_200_rp	ATGGTCTAGAAAGCTTTACATCTTCTTGTTGTTTGCGATGTAG

Figure S2

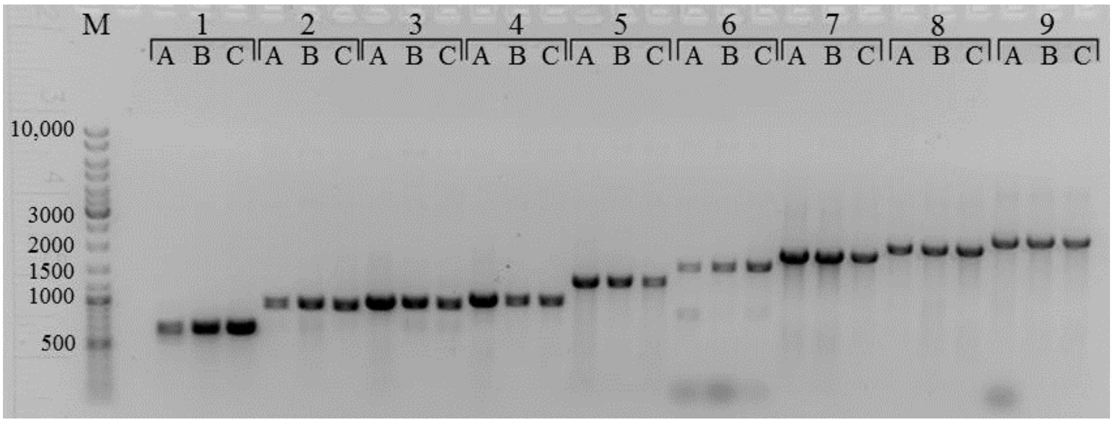

Fig. S2. PCR validation of cloned hCNTF in pOPIN vectors. M, **1-9** denote DNA marker and the set of expression vectors as listed in Table 1, respectively. A, B & C refer to randomly picked three colonies for positive clone screening.

Figure S3

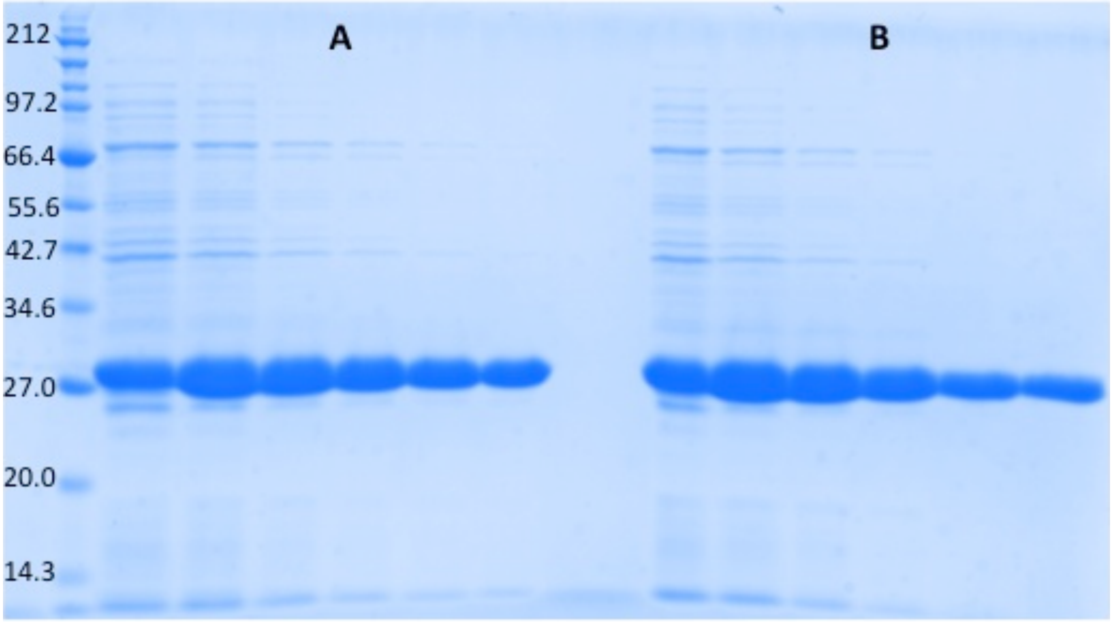

Fig. S3. Representative eluted fractions from Ni-IDA batch purification of 2 X 450 ml cultures (A and B).

Figure S4

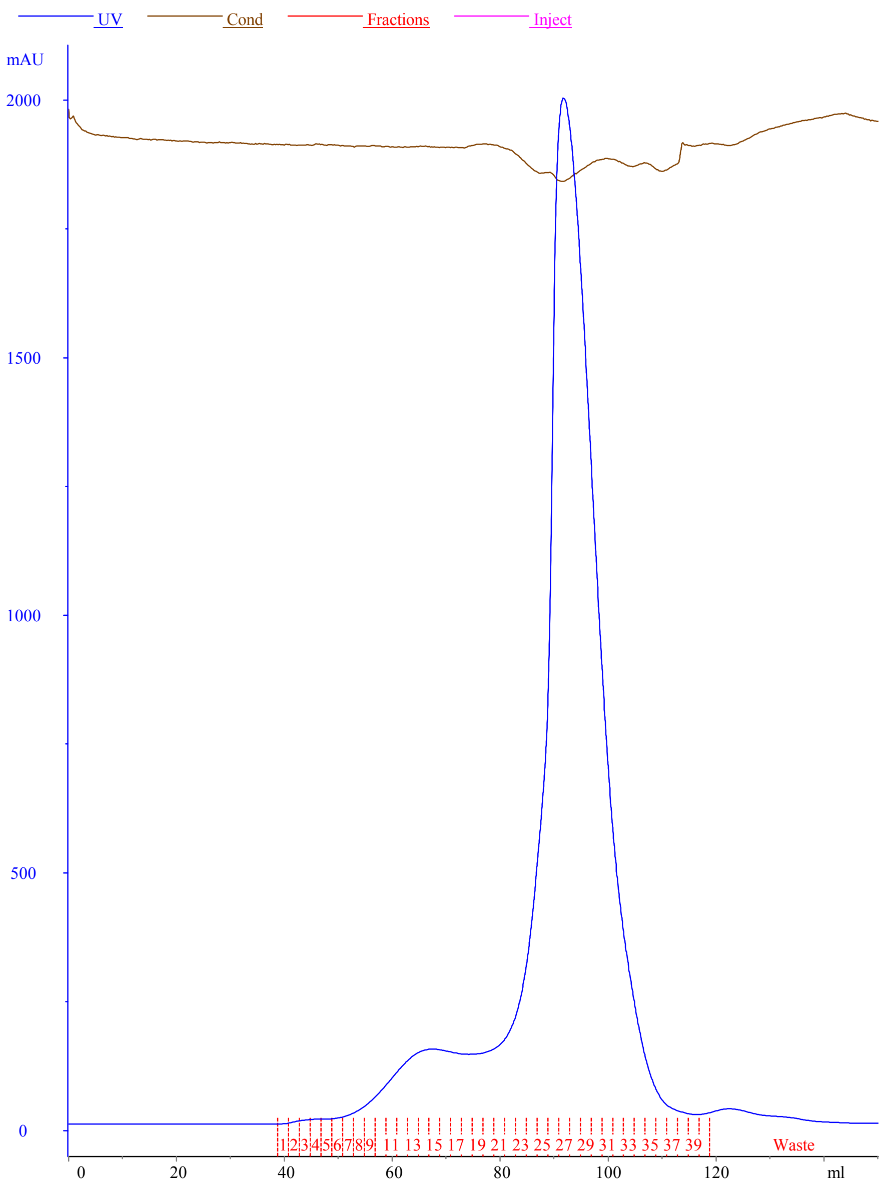

Fig. S4. Size exclusion chromatography (SEC). Chromatogram depicting eluted fractions from a HiLoad Superdex 200 gel filtration column run on AKTA purification system.
